# Thickness Effect on Structural, Electrical, and Optical Properties of Ultrathin Platinum Films

**DOI:** 10.3390/molecules30244794

**Published:** 2025-12-16

**Authors:** Roman R. Altunin, Evgeny T. Moiseenko, Ivan V. Nemtsev, Anna V. Lukyanenko, Mikhail V. Rautskii, Anton S. Tarasov, Valeriy S. Gerasimov, Oleg V. Belousov, Sergey M. Zharkov

**Affiliations:** 1 Siberian Federal University, 79 Svobodny Ave., 660041 Krasnoyarsk, Russia; e.t.moiseenko@ya.ru (E.T.M.); 2 Krasnoyarsk Scientific Center, Federal Research Center KSC SB RAS, Akademgorodok 50, 660036 Krasnoyarsk, Russia; ivan_nemtsev@mail.ru (I.V.N.); 3 Kirensky Institute of Physics, Federal Research Center KSC SB RAS, Akademgorodok 50/38, 660036 Krasnoyarsk, Russia; lav@iph.krasn.ru (A.V.L.); taras@iph.krasn.ru (A.S.T.); 4 Institute of Computational Modelling SB RAS, Akademgorodok 50/44, 660036 Krasnoyarsk, Russia; gerasimov@icm.krasn.ru (V.S.G.); 5 Institute of Chemistry and Chemical Technology, Federal Research Center KSC SB RAS, Akademgorodok 50/24, 660036 Krasnoyarsk, Russia; ov_bel@icct.ru (O.V.B.)

**Keywords:** platinum, ultrathin films, film thickness, structural properties, resistivity, optical transmission

## Abstract

Owing to the fact that ultrathin platinum films have many practical applications, the information concerning the initial stage of the formation of these films raises considerable interest. The effect of the film thickness on the morphology, as well as on the electrical and optical properties, was experimentally studied by a combination of methods (TEM, SAED, SEM, AFM, optical spectrophotometry, and electrical resistance measurements). The growth mechanisms of the films with an average thickness from 0.2 to 20 nm were determined, which is equivalent to the thickness of 1 to 100 monolayers (ML). The percolation threshold was reached, with the average film thickness being ≈1.0 nm, when electrical conductivity appeared. With an average thickness of ≈2.0 nm, the platinum films became almost continuous. The obtained data were analyzed within the framework of scaling theory. The growth of the platinum films at the initial stage (0.2–2.0 nm) was shown to proceed in the mixed 2D/3D growth mode. Here, 3D nanoislands, having a crystalline structure, were formed simultaneously with the formation of an almost continuous 2D subnanometer layer possessing an amorphous-like structure.

## 1. Introduction

Thin films, including those with a layer thickness smaller than 100 nm, have been widely used in microelectronics [1] and many other areas for a period of fifty years. Moreover, in recent years, the thickness of the produced films has steadily decreased, with the films fabricated now being about 1–10 nm thick; such films could already be called ultrathin. The physical properties of these films could be qualitatively different from the properties of thin films. This transfer to ultrathin layers has become possible, on the one hand, due to progress in the methods of obtaining films, which allow controlling the layer thickness at the atomic level, and on the other hand, due to the fact that these films are in high demand in different areas, such as nanoelectronics [2,3,4,5,6,7], optoelectronics [8,9,10], including photovoltaics [11,12,13], catalysis [14,15,16], sensors [17,18,19,20,21], etc.

Composition and fabrication conditions are known to predetermine the structure of a material, and the structure, in its turn, determines the physical properties of the material. There are three main mechanisms of the initial stage of near-equilibrium film growth: Frank–van der Merwe, Stranski–Krastanov, and Volmer–Weber [22,23,24,25]. The Frank–van der Merwe growth mode, which is also referred to as “layer by layer growth”, is two-dimensional (2D) growth, being observed in the case of growing epitaxial structures. The Stranski–Krastanov growth mode is a combination of two-dimensional (2D) growth at the initial stage (1–2 monolayers), followed by the growth of three-dimensional (3D) islands. And, finally, the Volmer–Weber growth mode is characterized by the growth of 3D islands, with the islands finally occupying the whole surface of the substrate, which results in the film becoming continuous. The above growth modes are universal and can be implemented under certain conditions, even during crystallization of amorphous films [26]. However, for each of these mechanisms to be observed, there must be well-defined conditions. The Frank–van der Merwe growth mode appears because the atoms of the deposited material are more strongly attracted to the substrate than to each other. In the opposite case, where the deposited atoms are more strongly bound to each other than to the substrate, the Volmer–Weber mode, with the formation of islands, is observed [22]. Also, for the Frank–van der Merwe or Stranski–Krastanov growth mode to be implemented, there must be a flat single crystal substrate heated to about 100–200 °C, and a certain energy of the atoms deposited on the substrate. The Volmer–Weber growth mode can be observed both with the substrate being a single crystal or an amorphous one. Here, in the case of the single crystal substrate, a coherent crystallographic orientation of islands can be observed, while in the case of the amorphous substrate, the islands are randomly oriented. It is worth noting that these classical mechanisms in the initial growth stage are implemented as long as surface diffusion transport is sufficient. When the atoms are not mobile enough, there begins the next stage (the kinetic mode), the formation of a polycrystalline or columnar structure [27]. The transition from the near-equilibrium mode to the kinetic one occurs when the clusters reach a critical size, which can be estimated using the calculation by Mullins [28].

Depending on the implemented mechanism of the thin film growth, it is possible to obtain various morphologies of thin films, from epitaxial layers to individual nanoparticles of the substrate surface. Moreover, in the nanostructures, both metastable and high-temperature phases can be formed, for example, HCP Au [29], hexagonal 4H-Ag [30], BCT Ag [31], HCP Pd [32], HCP Cu [31], BCC Cu [31], FCC Co [33,34], BCC Co [35,36], HCP Ni [37,38,39], and FCC carbon [40,41,42], as well as multiply-twinned particles [43,44,45]. Here, certain phases, for instance, HCP Pt, Ag, and Ir, can also be formed according to the density-functional simulations [46]; however, these phases have not been observed experimentally.

A decrease in the size of individual nanoparticles or nanocrystallites constituting the film can dramatically influence the physical properties and result in a considerable decrease in the melting temperature [47] or in a change in the optical or electrical properties. For example, some metals, in the form of nanoparticles or island films, show surface plasmon resonance (SPR) in the visible spectrum range: Au [48,49,50,51], Ag [52,53,54], Au-Ag [55], Ag-Pt [56,57], Cu [58], and Ni [59]. There are quite a few reports on the observation of SPR in Pt nanoparticles [56,60,61,62]. The increased interest in nanostructures having SPR is due to the fact that they can be used as sensors for detecting chemical and biological compounds [63].

As compared to other metals, platinum is rather attractive for many practical applications owing to its thermodynamic stability and kinetic inertness. Platinum in the form of nanoparticles, thin, and ultrathin films is of great interest in a large number of areas (nanoelectronics, catalysis, sensors, etc.), however, there are only few systematic studies on the effect of the platinum layer thickness on the morphology, phase composition, optical, and electrical properties in the range from several monolayers (for platinum 1 ML ≈ 0.2 nm) to several tens of monolayers. Films with a thickness of 1–2 ML are of special interest since, under certain conditions, they can possess semiconducting properties [15].

The present study aimed to investigate the growth mechanisms of ultrathin platinum films with an average thickness from 0.2 to 20 nm, obtained by magnetron sputtering. The investigation focused on the effect of the average platinum film thickness on the film morphology and size of the crystallites, on electrical resistivity, and optical transmission of the films in a wavelength range of 190–1100 nm. The thermal stability of the films and the effect of the heating temperature on the morphology and phase composition of the films, as well as on the value of electrical resistance, were also studied. The investigations were carried out using a combination of methods, including TEM, SEM, AFM, and optical spectrophotometry, as well as methods of measuring electrical resistance.

## 2. Results

### 2.1. The Structure and Morphology of the Pt Thin Films

#### 2.1.1. SEM, AFM

**Figure 1:**
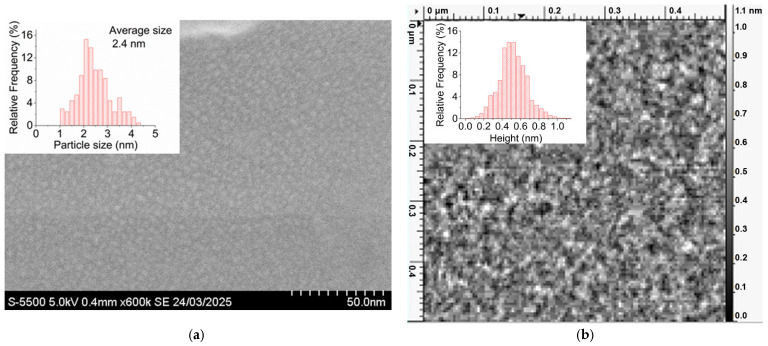
SEM image (**a**) and AFM image (**b**) obtained from a platinum film, with an average thickness of 0.5 nm. The insets show (**a**) a histogram of crystallite size distribution and (**b**) a height distribution histogram.

The study of the morphology of platinum thin films with an average thickness of 0.5 nm, deposited onto a non-conductive quartz substrate using the method of scanning electron microscopy (SEM), showed the films to consist of individual particles with a mean lateral dimension of 2.4 nm (Figure 1a), which is in agreement with the data presented earlier in [64]. Here, it is worth noting that the given SEM image (see Figure 1a) was obtained without depositing an additional conductive coating; however, the film provided a charge drain sufficient to prevent the sample from being charged. This evidence shows that, in spite of the fact that the given platinum films with a thickness of 0.5 nm consist of individual particles, there is an electrical contact between these particles.

The investigation of the 0.5 nm platinum films using the method of atomic force microscopy (AFM) showed the film thickness to be in the range from 0 to 1.1 nm, with the average film thickness being 0.5 nm (Figure 1b), which fully coincided with the target value. Moreover, it should be noted that areas with zero height, i.e., not filled with platinum, are almost absent in the AFM images (see Figure 1b).

The analysis of the SEM and AFM data shows the platinum films with an average thickness of ≈0.5 nm consist of individual platinum particles, with a lateral dimension of ≈2.4 nm and a height of ≈0.6–1.1 nm. In this case, the gaps between the particles are filled with a subnanometer layer of platinum, which, in particular, provides an electron drain sufficient for acquiring SEM images. However, it is to be noted that for the observed particle size, the resolution of the SEM method is no longer sufficient; therefore, further investigations were carried out by the method of transmission electron microscopy.

#### 2.1.2. TEM

In order to study the effect of the thickness on the morphology, electrical, and optical properties of ultrathin platinum films, films with the following average thicknesses were obtained: 0.2, 0.5, 0.7, 1.0, 1.4, 2.0, and 3.0 nm. The comparison of the results of the TEM investigation carried out using the platinum films with the thickness of 0.5 nm, deposited both on amorphous carbon films with a thickness of 15–20 nm and on SiO_2_ films with a thickness of 15–20 nm, showed the morphology of the films to be similar to that of the 0.5 nm platinum films deposited on a quartz substrate (see Section 2.1.1). In particular, the film consists of crystallites with an average lateral size of ≈2.4 nm. The analysis of the SEM, AFM, and TEM data allows us to assume the gaps between the crystallites to be filled with a subnanometer amorphous-like platinum layer, with a thickness of <0.6 nm (i.e., smaller than 3 monolayers), and the films seem to consist of individual crystallites.

As concerns the morphology of the films on various substrates, the authors in [65] earlier showed Pt and Ni films deposited on glass and amorphous carbon to have the same morphology, which confirms the results obtained in the present study. Similar results were obtained for Pd films [66]. In this study, use was made of electron microscopy grids covered with a layer of amorphous carbon since they, unlike SiO_2_, allow sample heating. In principle, an experimental study of the cross-sectional structure of ultrathin platinum films by HRTEM could confirm the film morphology proposed above. However, conducting such a study is significantly complicated by the fact that the thickness of the amorphous-like platinum layer is only 1–2 monolayers. Therefore, all the studies of the ultrathin platinum films by the TEM method were carried out in plan-view geometry.

**Figure 2:**
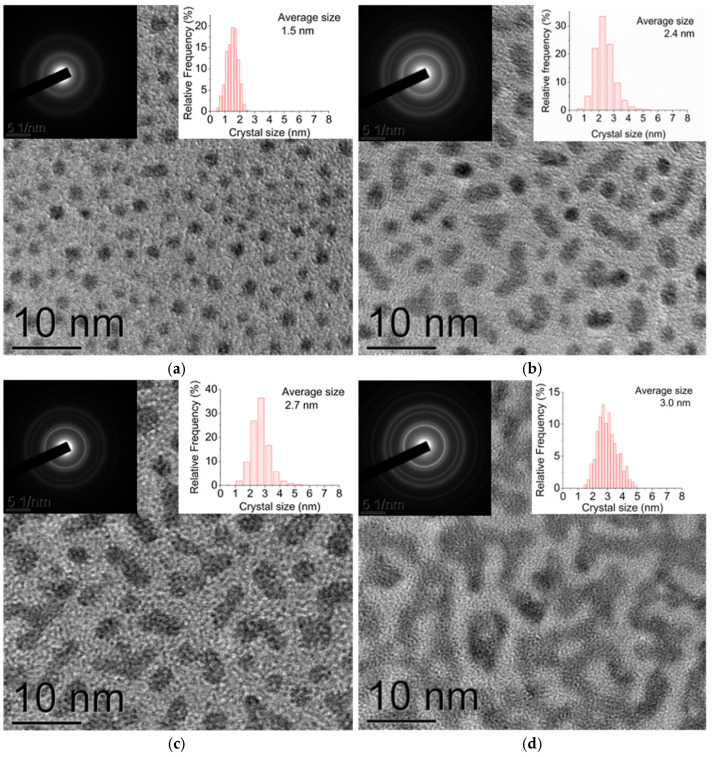

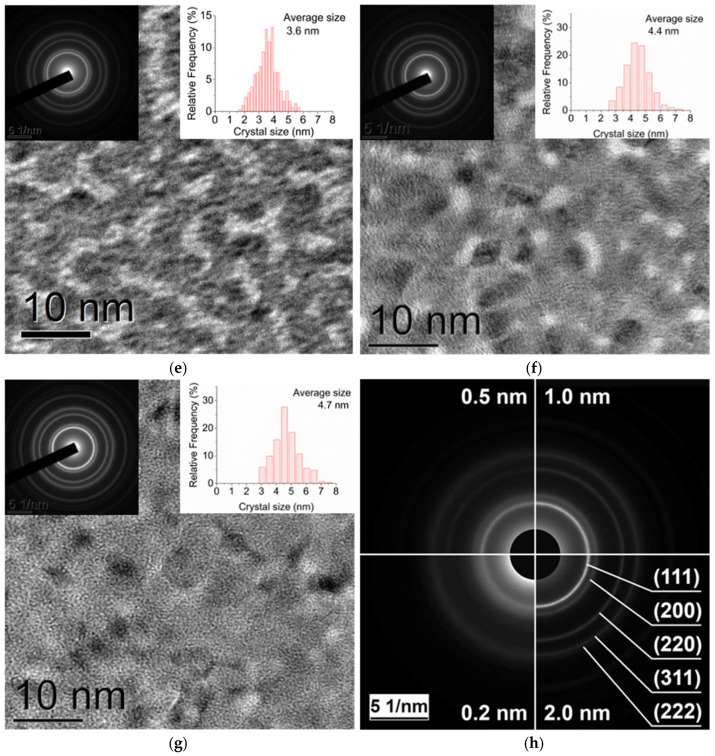
Plan-view TEM images of the Pt film with different average thicknesses: (**a**) 0.2 nm; (**b**) 0.5 nm; (**c**) 0.7 nm; (**d**) 1.0 nm; (**e**) 1.4 nm; (**f**) 2.0 nm; and (**g**) 3.0 nm. The insets in (**a**–**g**) show the corresponding SAED patterns and histograms of crystallite size distribution. The fragments of SAED patterns obtained from the films with thicknesses of 0.2, 0.5, 1.0, and 2.0 nm (**h**).

The analysis of the TEM images (Figure 2a–g) allowed estimating the size of the platinum crystallites and the fractional surface coverage for different average thicknesses of the platinum films, from 0.2 to 3.0 nm (Table 1). As is seen from Table 1, with an increase in the film thickness, one can observe both an increase in the average crystallite size and in the fractional surface coverage. For example, for the 0.5 nm films, the fractional surface coverage amounted to 31%, with the individual crystallites beginning to combine to form islands. In the case of the film thickness of 2.0 nm, the fractional surface coverage was 95%, while it was 100% in the case of the 3.0 nm thickness. The data obtained in the present study correlate with the data from other authors, for example, in [63,67], based on the analysis of the dependence Rt^2^(t) (here R is the electrical resistance, and *t* is the film thickness), and a calculation was performed which shows that the platinum film became continuous upon reaching thicknesses of 2.3 and 3.9 nm, respectively. In [68], the TEM investigation showed that the film became continuous with a thickness of 4.0 nm.

It is worth noting that the analysis of the electron diffraction patterns obtained from the area of 1.3 μm (Figure 2h) shows that in all the cases the crystal lattice corresponds to the fcc phase of platinum. Moreover, the lattice constant observed, a = 3.92 ± 0.02 Å, corresponds to the lattice constant of platinum in the bulk state (PDF 4+ card #04-011-782, Fm-3 m, a = 3.913 Å). The analysis of the diffraction reflection intensity shows the platinum crystallites in the films with a thickness from 0.2 to 3.0 nm to be randomly oriented. Here, the changes in the film thickness result only in the changes of the width of the diffraction reflections due to the changed size of the crystallites, and it has no impact on the location of the diffraction reflections (see Figure 2h).

In order to study the effect of heating on the morphology, platinum films with a thickness of 1.4 nm were heated in the column of a transmission electron microscope to 300 °C at a rate of 5 °C/min, with subsequent annealing at 300 °C for 15 min, followed by cooling to room temperature. The analysis of the morphology of the 1.4 nm Pt films showed that the cycle, which included heating, annealing, and cooling, had no impact on the morphology and phase composition of the films.

### 2.2. Optical Properties of the Pt Thin Films

**Figure 3:**
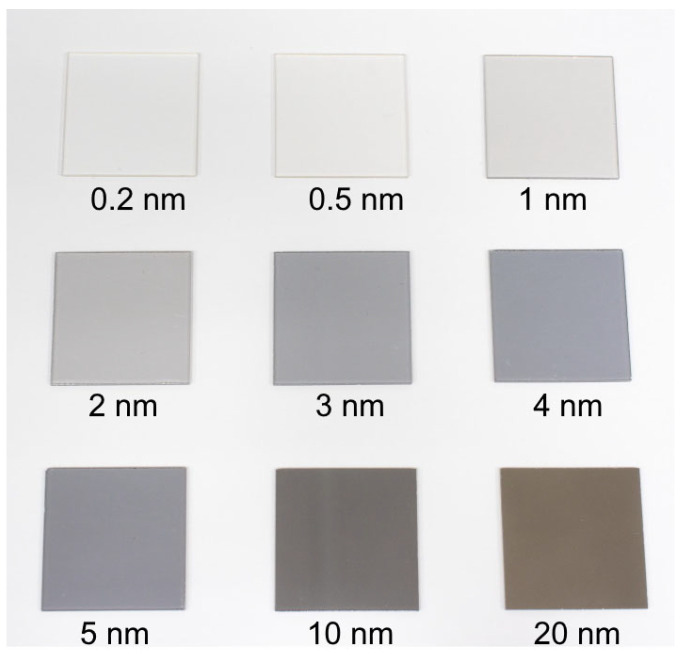
Images of the platinum thin films with different thicknesses (0.2–20 nm).

A series of Pt films with thicknesses of 0.2–20 nm was obtained in order to investigate the dependence between the Pt film thickness and transmission spectra. The general layout of the obtained platinum films with various thicknesses is presented in Figure 3.

**Figure 4:**
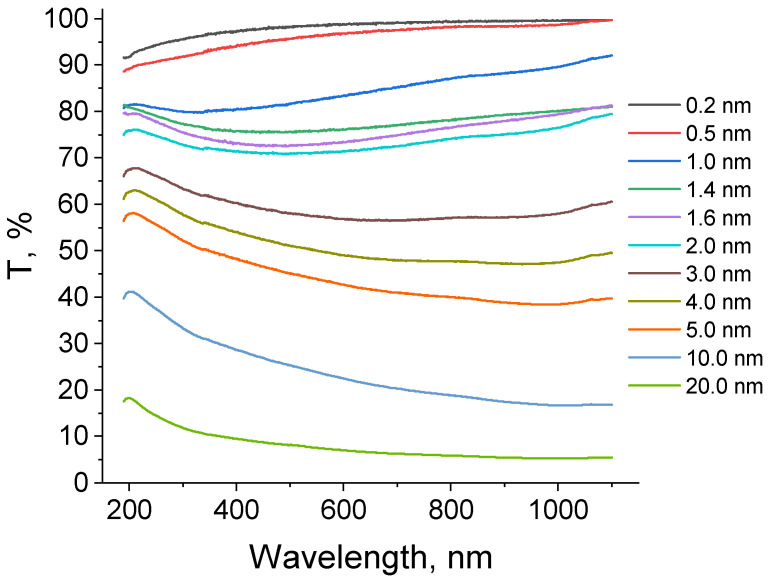
Optical transmission spectra of the platinum thin films of different thicknesses.

Figure 4 presents the transmission spectra of the platinum thin films with different thicknesses in a range of 190–1100 nm, with the corrections made taking into account the absorption by the quartz substrates.

There is a strong correlation between the film thickness and the value of optical transmission. As is expected, transmission in the visible spectrum range decreases monotonically with an increase in the film thickness. The ultrathin films (0.2–0.5 nm) demonstrate high transparency, typical for a discontinuous insular structure in which the substrate is partially uncovered. A significant drop in the transmission in the range from 1.0 to 2.0 nm indicates the formation of a percolated layer. This is in agreement with the measurements of electrical resistance discussed in Section 2.3.1. For the thicker films (3–20 nm), the transmission curves correspond to the typical behavior of a continuous metal film, demonstrating lower values of transmission. No noticeable features of SPR were found. The most probable reason for this is that the size of the isolated nanoparticles during the initial stages of platinum film growth is only 1.5–2 nm. Such small particles do not exhibit a pronounced plasmon resonance, as the mean free path of conduction electrons significantly exceeds the particle size itself, and the plasmon resonance is suppressed due to a large relaxation constant associated with electron scattering at the surface. Typically, plasmon resonance is observed in nanoparticles larger than 10 nm. For instance, in the case of island gold films, the average island diameter ranges from 8 to 100 nm [51]; for silver films, from 25 to 100 nm [52]; and finally, for platinum, from 40 to 100 nm, with the height of the platinum islands ranging from 10 to 30 nm [62].

### 2.3. Electrical Properties of the Pt Thin Films

#### 2.3.1. The Effect of Pt Film Thickness on the Value of Electrical Resistivity

**Figure 5:**
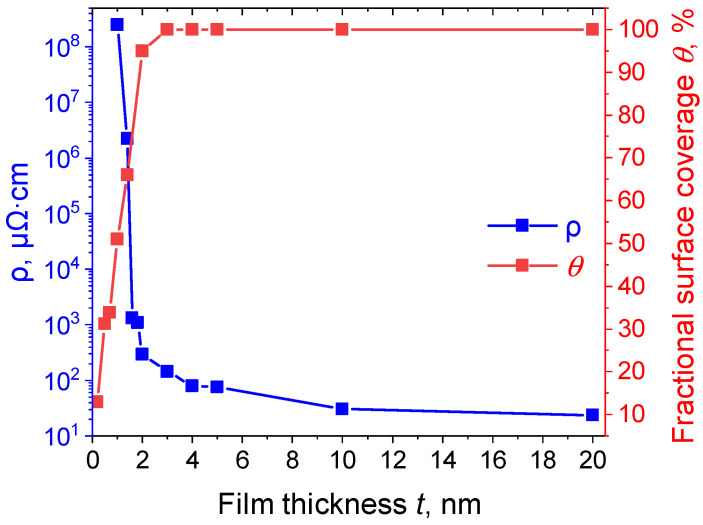
Dependence of the resistivity ρ (blue line) and the fractional surface coverage *θ* (red line) on the Pt film thickness *t*.

To determine the dependence between the average thickness of the platinum films and their electrical properties, electrical resistance was measured by the four-probe method. The average thicknesses of the Pt films amounted to 0.2, 0.5, 0.7, 1.0, 1.4, 2.0, 3.0, 4.0, 5.0, 10, and 20 nm. Figure 5 presents the dependence of the electrical resistivity and the fractional surface coverage on the Pt film thickness.

The percolation threshold (the thickness at which the film becomes conductive) in the platinum films is determined to be reached upon achieving a thickness of 1.0 nm, which is in good agreement with the data obtained earlier, namely 0.83 nm in [63] and 1.3 nm in [65]. In this case, the value of the electrical resistivity for a film thickness of 1.0 nm is equal to ρ = 2.52∙10^8^ μΩ∙cm. Witssh an increase in the film thickness and, consequently, in the fractional surface coverage, one can observe an abrupt decrease in electrical resistivity (see Figure 5) to a thickness of 3.0 nm (ρ = 142.8 μΩ∙cm), which is due to the changes in the film morphology. As shown in Section 2.1.2, at a thickness of 3.0 nm, the films become continuous. Moreover, with the film thickness exceeding 3.0 nm, the size of the crystallites changes only insignificantly, resulting in a slight decrease in the electrical resistivity. The value of the electrical resistivity ρ = 30.6 μΩ∙cm for the films with a thickness of 10 nm is in good agreement with the data obtained in other studies (ρ = 43.5–72.5 μΩ∙cm [12,13]). In addition, the electrical resistivity of the platinum films with a thickness of 20 nm ρ = 23.6 μΩ∙cm is comparable to the values typical for platinum in the bulk state (ρ = 10.5 μΩ∙cm at 20 °C [69]).

#### 2.3.2. Temperature Dependence of the Electrical Resistance on Platinum Island Films

**Figure 6:**
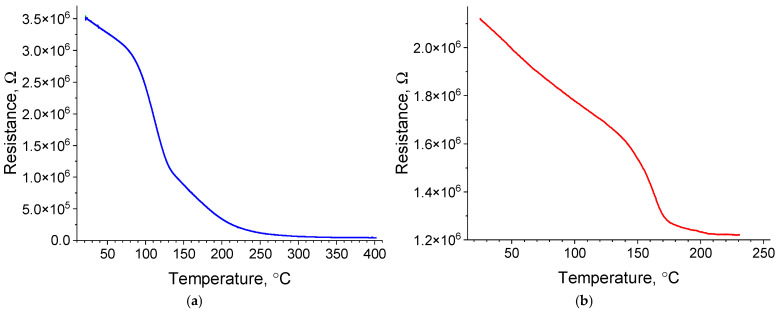
Dependence of the electrical resistance on the temperature of the 1.4 nm thick Pt films upon heating in a vacuum of 1 × 10^−6^ Torr (**a**) and in air (**b**).

The temperature dependence of the electrical resistance of 1.4 nm thick island Pt films was studied. The films were heated from room temperature to 400 °C under high vacuum–1 × 10^−6^ Torr (Figure 6a) and to 230 °C in air (Figure 6b). The heating rate in both cases was 5 °C/min. The electrical resistance of the films was found to decrease during heating, both in the vacuum and in air, which is known to be atypical for the temperature dependence of the electrical resistance in metals. At the same time, while in the case of heating from room temperature to 230 °C in the vacuum, the resistance decreased from 3.55 × 10^6^ Ω to 1.76 × 10^5^ Ω. Upon heating in air in the same temperature range, the resistance decreased from 2.14 × 10^6^ Ω to 1.22 × 10^6^ Ω. The difference in the absolute value of the initial resistance is due to the use of different measurement methods. The four-probe method was used for heating in the vacuum, while the two-probe method was used for heating in the air.

Taking into account the morphology of the films, a number of explanations can be suggested for the observed dependence: a change in the size of crystallites; a decrease in the degree of film defects; dissociation of platinum oxides; and a semiconductor temperature dependence.

The electrical resistance of metal films is known to be inversely proportional to the crystallite size of these films [70,71]. Moreover, it is known that heating the films can lead to an increase in the crystallite size due to diffusion. However, as noted in Section 2.1.2, heating and annealing the 1.4 nm thick Pt films up to 300 °C do not affect the crystallite size or the fractional surface coverage. Thus, the observed dependence of the electrical resistance cannot be explained by a change in the crystallite size.

Heat-induced grain boundary diffusion can reduce film defectivity by healing it [72]. This reduction in the film defectivity, in turn, leads to a decrease in electrical resistance. However, the methods used in this study do not allow one to estimate changes in the defect structure during film heating. Therefore, the effect of this mechanism on the temperature dependence of the electrical resistance of ultrathin platinum films cannot be ruled out.

If, during the production process and/or after removal from vacuum, oxygen formed platinum oxides at the crystallite boundaries of thin Pt films, this could have led to an increase in the electrical resistance of the films in their initial state. Dissociation of these oxides as a result of heating would lead to a decrease in the electrical resistance of the platinum films. Table 2 presents the estimation results of the onset temperature of platinum oxide dissociation, obtained using the Van’t Hoff isotherm equation for different air pressures.

As can be seen from Table 2, the calculated dissociation temperature of platinum oxides under high vacuum conditions at a pressure of 1 × 10^−6^ Torr (112 °C) corresponds to the region of a sharp decrease in electrical resistance when heating the films in a vacuum (see Figure 6a). However, in the case of heating in air, the heating temperature is insufficient (≤430 °C, see Table 2) for the dissociation of oxides, which leads to the resistance decreasing less significantly in this case.

Some platinum oxides (in particular, PtO_2_ [73]) are known to be semiconductors, which could account for the observed temperature dependence on the electrical resistance (see Figure 6a). However, in the present study, platinum oxides could only have formed in small quantities at the boundaries of platinum crystallites; thus, their amount could not significantly affect the nature of the temperature dependence on the resistance of platinum thin films.

Thus, having considered all possible explanations, it can be assumed that in the case of heating platinum thin films under high vacuum conditions, the main mechanism determining the decrease in electrical resistance during heating in a vacuum is the dissociation of platinum oxides, along with a decrease in film defectivity due to diffusion, whereas in the case of heating in air, the main mechanism is a decrease in the film defectivity.

## 3. Discussion

**Figure 7:**
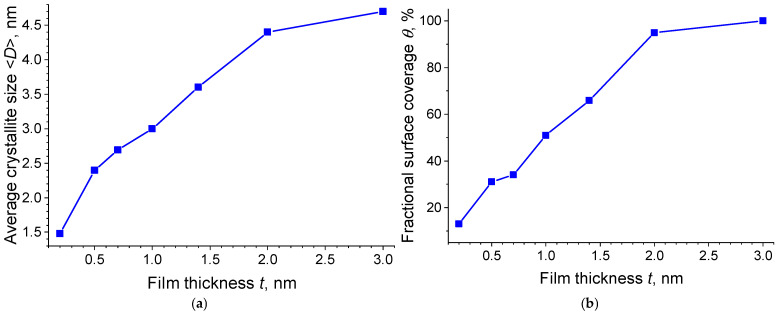
Dependence of the crystallite size <*D*> (**a**) and the fractional surface coverage *θ* (**b**) on the Pt film thickness *t*.

The analysis of the data obtained by TEM (see Table 1) shows that the dependence of the crystallite size and the fractional surface coverage on the Pt film thickness has a power-law character (Figure 7a,b), which, in turn, suggests the presence of scaling during the growth of the Pt films. According to the model by Blackman and Wilding [74], the dependences of the fractional surface coverage *θ*, average crystallite size <*D*>, and island density *N* on the film thickness *t* can be described with the power functions: *θ*~*t*^α^, <*D*>~*t*^β^, and *N*~*t*^γ^. Here, the analysis of the power coefficients α, β, and γ can be used to obtain information about the film growth mode.

A similar analysis is performed in [68] for Au, Cu, and Pt thin films based on the results obtained by TEM. In particular, gold and copper films were found to grow in the 3D mode at the aggregation stage. In the case of platinum, at the aggregation stage, the films were assumed to grow in the 2D mode. However, it should be noted that for platinum films, the authors in [68] analyzed only the dependence of the fractional surface coverage *θ* on the film thickness *t* and did not consider the dependences of the average island size and island density on the film thickness, which, in turn, could lead to an incorrect interpretation of the obtained results. It should be noted that in [75,76,77,78], platinum films on various substrates, TiO_2_ (110), Al_2_O_3_ (0001), a-C, and a-SiO_2_, were stated to grow in the 3D mode. The analysis of the aforementioned works shows that the conditions for the deposition of platinum island films varied within a fairly wide range. For instance, in [75], platinum was deposited by thermal evaporation at a rate of 0.05 nm/min, and the temperature of the single-crystal TiO_2_ (110) substrate varied from −113 to +147 °C. Based on the analysis of low-energy ion scattering spectroscopy data, the authors of [75] conclude that platinum grows in the Volmer–Weber growth mode with a (111) orientation. In [76], platinum films were obtained by magnetron sputtering at a deposition rate of 1.7 nm/min. A single-crystal Al_2_O_3_ with a (0001) orientation was used as the substrate; the substrate temperature during the deposition was 650–750 °C, and the platinum films grew in the 3D mode with a (111) orientation. In work [77], platinum films were obtained by plasma deposition at a rate of 0.25 nm/min, using amorphous carbon films and a-SiO_2_/Si (100)—a monocrystal covered with a 2 nm thick native oxide layer—as substrates. Based on the ratio of the island diameter to their height, it can be concluded that 3D islands form during the growth of platinum films. However, it should be noted that the island height was determined using the GISAXS method, the resolution of which is significantly limited for structures with a height of less than 1 nm. Meanwhile, as shown in the present work (see Section 2.1.2), the space between the platinum islands is filled with an amorphous subnanometer layer with a thickness of less than 0.6 nm. Therefore, the characterization method used in [77] prevented the authors from detecting the presence or absence of the subnanometer layer. In [78], thin Pt films were deposited by magnetron sputtering onto a polycrystalline WO_3_ film at room temperature. The platinum deposition rate was 12 nm/min. The authors suggested that platinum nanoparticle formation (the average size ≈ 2.5 nm) occurs via the Volmer–Weber type growth mode.

The only study where the authors conclude that platinum films initially grow in the 2D mode is work [68]. In that study, platinum films with a thickness of 0.2–2 nm were obtained by ion beam sputtering. Amorphous carbon substrates with a thickness of 20 nm, held at room temperature, were used. The platinum deposition rate was 0.1–0.2 nm/min (these data are provided in an earlier work by the same authors [79]). Based on the analysis of scaling dependences in [68], it was suggested that the platinum islands grow as flat two-dimensional islands (i.e., they grow much faster in the lateral dimensions than in the vertical (thickness) direction).

It should be noted that it was the analysis of scaling relationships, which had not been conducted in other studies, that allowed the authors of [68] to suggest the presence of the 2D growth mode during the initial stage of ultrathin platinum film formation. In order to determine the growth mode (2D or 3D) of platinum films at the initial stage, in this study, the scaling dependencies of the fractional surface coverage *θ*, the average crystallite size *<D>*, and the island density *N* on the film thickness were analyzed.

### 3.1. Analysis of the Dependence of the Fractional Surface Coverage on the Pt Film Thickness

**Figure 8:**
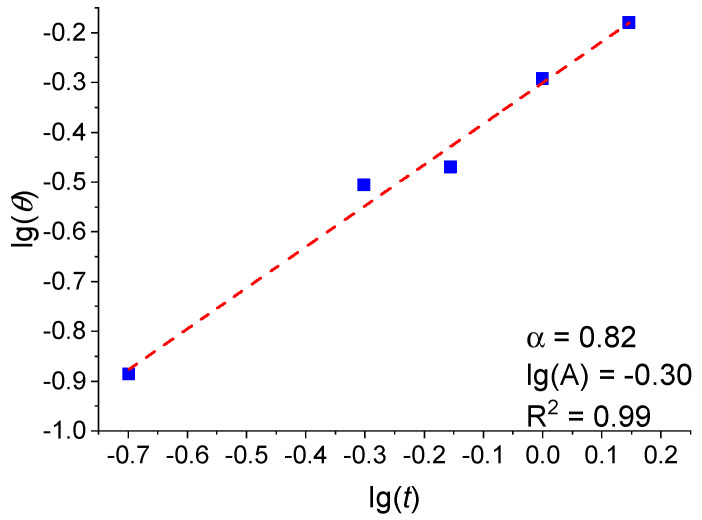
Dependence of the fractional surface coverage *θ* on the Pt film thickness *t* in logarithmic coordinates.

Figure 8 presents the dependence of the fractional surface coverage *θ* on the Pt film thickness *t* in logarithmic coordinates. As is seen in Figure 8, the experimental data are well described by the power function *θ* = *A∙t*^α^, where α = 0.82, *A* = 0.50. Here, the power coefficient α shows how efficiently the fractional surface coverage increases with the increasing film thickness. *A* is a constant, depending on the fabrication method.

According to the Blackman–Wilding model [72] for the aggregation stage, and in the absence of dissociation, the value of the coefficient α ≈ 1 corresponds to the 2D growth mode of islands, with the material being uniformly distributed over the substrate. The value α ≈ 0.7 corresponds to the 3D growth mode, when the material mainly increases the height of existing islands [72]. In the present study, α = 0.82, which corresponds to an intermediate value between the theoretically calculated values for ideal 2D/3D growth modes at the aggregation stage. The analysis of the data obtained by the SEM, TEM, and AFM methods (see Section 2.1.1 and Section 2.1.2) indicates the simultaneous formation of both 3D islands and a 2D subnanometer layer filling the space between them, which is confirmed by the value of the coefficient α = 0.82. Moreover, the analysis of the TEM images (see Figure 2) shows that as early as an average thickness of 0.5 nm, individual crystallites begin to coalesce to form islands. Thus, it can be assumed that the growth process of Pt films occurs via a mixed mechanism, including both aggregation and coalescence.

### 3.2. Analysis of the Dependence of the Crystallite Size on the Pt Film Thickness

**Figure 9:**
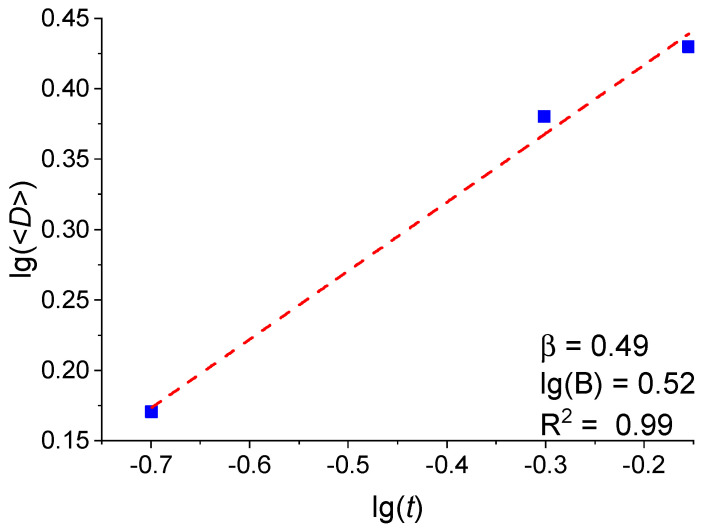
Dependence of the average crystallite size on the Pt film thickness on a logarithmic scale.

To analyze the average crystallite size and island density, use was made of the data obtained from the films with thicknesses of 0.2, 0.5, and 0.7 nm, since isolated islands are observed only at these thicknesses. Figure 9 shows the dependence of the average crystallite size <*D*> on the Pt film thickness *t* in logarithmic coordinates. As is seen in Figure 9, the experimental data are well described by the power function *<D>* = B*t*^β^, where β = 0.49 and B = 3.31. Here, β shows how efficiently the crystallite size grows with the increasing film thickness. B is a constant, depending on the fabrication method.

The obtained coefficient β = 0.49 is significantly lower than the calculated values β ≈ 0.86 for the 3D growth mode and β ≈ 1 for the 2D one [72]. According to the Blackman–Wilding model, the decrease in β may be caused by dissociation. However, for β = 0.49, the calculations of the critical cluster size *m* for the case with dissociation have no physical meaning (the calculated value *m* < 1 for the 2D growth mode and *m* < 0 for the 3D one). This inconsistency can be resolved within the framework of the models, which take into account the contribution of the Schwoebel barrier [80,81], which hinders the attachment of atoms to the boundaries of growing islands. In this case, the coefficient β can be lower than the values characteristic of both the 3D growth mode (β ≈ 0.86 [72]) and the 2D growth mode (β ≈ 1 [72]). The presence of this barrier in the Pt films studied here is confirmed by the AFM data (see Section 2.1.1), which indicate the presence of a subnanometer platinum layer filling the space between the islands. Thus, the process of crystallite growth with an increase in the thickness of platinum films obeys the scaling law, and the presence of the Schwoebel barrier leads to a slowdown in the growth of crystallites.

### 3.3. Analysis of the Dependence of the Island Density on the Pt Thin Films

**Figure 10:**
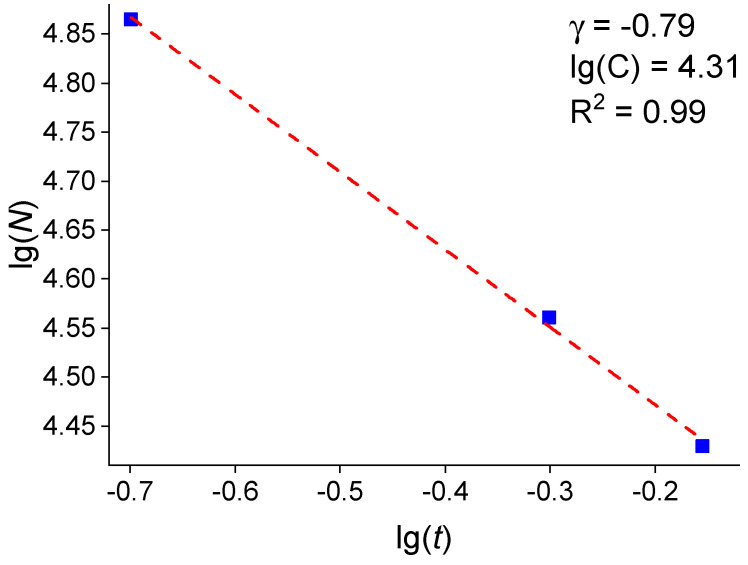
Dependence of the island density *N* on the Pt film thickness *t* in logarithmic coordinates.

Figure 10 presents the dependence of the island density *N* on the Pt film thickness *t* in logarithmic coordinates. As is seen in Figure 10, the experimental data are well described by a power function *N* = *Ct*^γ^, where *C* = 20,417 and γ = −0.79. Here, γ shows how efficiently the island density increases with an increasing film thickness, and *C* is a constant, depending on the fabrication method.

According to Blackman–Wilding [73], in the case of aggregation, the power coefficient is positive (γ > 0). However, in the present study, this coefficient has a negative value, γ = −0.79. Presented in [82] are the results of a study of island formation and coalescence during the room-temperature vapor phase deposition of aluminum using the Family–Meakin model [83]. In [78], the observed decrease in the island density during the deposition of aluminum is shown to be due to coalescence, and this is characterized by a negative power coefficient, γ = −2. Thus, the negative coefficient value γ = −0.79, obtained in the present study, confirms the assumption (see Section 3.1) that the growth process of Pt films occurs by a mixed mechanism, including both aggregation and coalescence.

Thus, it has been established that platinum films initially grow via a mixed 2D/3D mechanism, characterized by a power-law exponent α = 0.82. The following explanation for the observed mixed 2D/3D growth mechanism can be proposed. At the initial growth stage, nucleation centers for 2D structures form. Subsequently, the area of these structures increases. Upon reaching a critical nuclei size (0.6 nm or 3 ML in height), the growth transitions to the 3D crystallite growth mode. This 3D growth is limited by the Schwoebel barrier [80,81] and is characterized by a power-law exponent β = 0.49. A similar mechanism was proposed by the authors of [84] for the growth of thin metal films on weakly-interacting substrates (such as a-C, a-SiO_2_, and mica). Furthermore, this work establishes that the growth mechanism of platinum films involves both aggregation and coalescence, characterized by a negative power-law exponent γ = −0.79. It can be assumed that the described mechanism for thin platinum film growth will be realized on any amorphous substrate.

## 4. Materials and Methods

### 4.1. Method and Conditions of Fabricating Thin Film Samples

Platinum films were obtained by pulse DC magnetron sputtering using an EPOS-PVD-D-CONFOCAL high-vacuum setup, equipped with four 2-inch diameter round magnetrons ONYX (Angstrom Sciences, Duquesne, PA, USA). The base vacuum was 1 × 10^−6^ Torr, and the working vacuum (argon atmosphere) was 3 × 10^−3^ Torr. The argon purity was 99.999%, with the platinum target being 99.99 wt.% pure. The power supplied to the magnetron amounted to 11.5 W/inch^2^, with the deposition rate being 0.10 nm/s. The deposition rate and the deposited layer thickness were controlled using an INFICON (Bad Ragaz, Switzerland) SQC-310 thin film deposition controller. The average platinum film thicknesses ranged from 0.2 to 20 nm, which is equivalent to a thickness of 1 to 100 monolayers (ML). High-quality amorphous JGS1 Quartz Fused Silica (25 × 25 mm) and amorphous carbon films (15–20 nm) deposited on TEM grids were used as substrates. The substrate temperature during deposition was equal to room temperature. The stage with the substrates mounted on it rotated at a speed of 10 rpm during the film deposition.

### 4.2. Methods for Studying Structural Properties

#### 4.2.1. SEM and AFM

The morphology of films on the surface of JGS1 Quartz Fused Silica was studied using a Hitachi-5500 FE-SEM (Tokyo, Japan), with a resolution of 0.4 nm. The studies were conducted at an accelerating voltage of 5 kV. No conductive coating was deposited.

The AFM investigation was performed using NanoInk’s DPN 5000 System in the semi-contact mode. For the research cantilevers, NSG10 (Tips Nano, Tallinn, Estonia) were used.

#### 4.2.2. TEM and ED

The morphology and phase composition of the films were studied by TEM and selected area electron diffraction (SAED), using a JEOL (Tokyo, Japan) JEM-2100 (LaB_6_) at an accelerating voltage of 200 kV. Heating the thin-film samples directly in the electron microscope column allowed for studying the effect of temperature on the morphology and phase composition of the films. The conditions and features of in situ studies with heating in the TEM column are described in detail in our previous papers devoted to the study of solid-state reactions initiated by thermal heating in various thin-film systems: Al/Pt [85,86], Al/Au [87], Al/Ag [88], Al/Cu [89,90], Al/Fe [91], Fe/Pd [86,92], Cu/Au [93], Cu/Si [94], Co-ZrO_2_ [95], Al/a-Si [96], and a-Si crystallization [97].

### 4.3. Study of Electrical Properties

Electrical properties were measured by the 4-probe and 2-probe methods, using a Keithley 2450 Source Measure Unit (Solon, OH, USA). The measurements were performed on samples placed on a JGS1 Quartz Fused Silica substrate. The sample size was 25 × 25 mm. The effect of temperature on the electrical resistance was also investigated. The studies were conducted in the temperature range of 20–400 °C. The samples were heated both under high vacuum (1 × 10^−6^ Torr) and at atmospheric pressure.

### 4.4. Study of Optical Properties

Optical transmission spectra were measured using a SILab (Beijing, China) u-Violet DB spectrophotometer, designed to obtain absorption/transmission spectra of condensed matter in the 190–1100 nm range. The studies were performed on films placed on JGS1 Quartz Fused substrates measuring 25 × 25 mm. JGS1 has no absorption band in the 185–2500 nm range, which allows one to obtain information on the absorption/transmission spectrum over the entire wavelength range studied in this work.

## 5. Conclusions

The analysis of the thickness effect on the electrical resistivity showed the percolation threshold in the platinum films to be reached at a thickness of 1.0 nm (ρ = 2.52∙10^8^ μΩ∙cm). With an increase in the film thickness to 3.0 nm, the films became continuous, with the resistivity decreasing abruptly down to ρ = 142.8 μΩ∙cm. At a film thickness equal to 20 nm, ρ = 23.6 μΩ∙cm, which is only 2.25 times higher than the ρ value typical for the bulk platinum.

The analysis of the optical properties of the platinum thin films showed a strong correlation between the film thickness and the value of optical transmission. With a thickness of 0.2 nm, the transmission coefficient in the visible wavelength range was close to 100%, and with a thickness of 20 nm, it became lower than 10%.

Within the whole range of the thicknesses under study (0.2–20 nm), the growing platinum crystallites had the fcc structure typical for the bulk platinum.

As a result of the investigation carried out by the SEM and AFM methods, as well as following the TEM data analysis within the framework of scaling theory, it was found that at the initial stage (0.2–2.0 nm) the platinum films grew in the mixed 2D/3D growth mode, wherein the 3D growth of the islands is limited by the Schwoebel barrier and involves both aggregation and coalescence. The growth process of the platinum thin films was shown to be characterized by the following power coefficients within the Blackman–Wilding model: α = 0.82 for the fractional surface coverage, β = 0.49 for the average crystallite size, and γ = −0.79 for the island density. The value α = 0.82 obtained in this work is, on the one hand, significantly lower than the value of the coefficient α ≈ 1, which corresponds to the 2D growth mode of islands, and on the other hand, substantially higher than the value α ≈ 0.7, which corresponds to the 3D growth mode. Consequently, it is shown that the α value, which is intermediate between those characteristic of the 2D/3D growth modes, characterizes a mixed 2D/3D growth mode.

The film morphology was represented by 3D crystalline nanoislands and a 2D amorphous-like subnanometer layer filling the space between the islands. In spite of the presence of the 3D and 2D structures, the given growth mode cannot be considered Stranski–Krastanov, in which the 2D growth is followed by the 3D growth. In the present study, the simultaneous formation of the 3D nanoislands (similar to the Volmer–Weber growth mode) and of the 2D subnanometer layer growing in the non-equilibrium kinetic mode occurred. Since the substrate temperature was equal to room temperature, the surface mobility was insufficient to maintain the stationary Volmer–Weber mode. A significant number of atoms did not participate in the formation of the crystalline 3D nanoislands but remained in the 2D layer, which had an amorphous structure. With the film thickness larger than 2.0 nm, the growth occurred in the polycrystalline 3D mode, with the platinum crystallites oriented randomly.

The following results obtained in this work may have practical applications: the percolation threshold was determined, the thickness at which the platinum film became continuous was established, and the power-law coefficient α (effectively the fractional surface coverage) was estimated. The value of the coefficient α = 0.82 obtained in this work is higher than the known literature data for gold (α = 0.64) and copper (α = 0.71) [72], which means that the platinum film becomes continuous at a lower thickness than gold and copper films. This information can be useful for the application of platinum films as ultrathin electrical contacts.

It can be assumed that the mixed 2D/3D growth mode observed in this work will be characteristic of the initial stage of platinum film growth when sputtered onto any amorphous substrates at room temperature. Such films will possess a developed surface, making them more attractive for catalytic coatings than films grown using the Frank–van der Merwe growth mode.

## Figures and Tables

**Table 1:** Morphology of the Pt thin films of different thicknesses.

Average Pt Film Thickness *t*, nm	Average Crystallite Size <*D*>, nm	Fractional Surface Coverage *θ*, %
0.2	1.5	13
0.5	2.4	31
0.7	2.7	34
1.0	3.0	51
1.4	3.6	66
2.0	4.4	95
3.0	4.7	100

**Table 2:** Calculated dissociation temperatures of platinum oxides, depending on pressure.

Reaction	Air Pressure, Torr					
	770	77	0.77	7.7 × 10^−3^	5 × 10^−6^	1 × 10^−6^
	Onset Temperature of the Reaction, °C					
2PtO = 2Pt + O_2_	480	411	305	227	155	138
PtO_2_ = Pt + O_2_	430	366	267	195	128	112

## Data Availability

Data are contained within the article.

## Abbreviations

The following abbreviations are used in this manuscript:

AFM	Atomic Force Microscopy
BCC	Body-Centered Cubic
BCT	Body-Centered Tetragonal
ED	Electron Diffraction
GISAXS	Grazing incidence-small angle X-ray scattering
HCP	Hexagonal Close-Packed
ML	Monolayer
SAED	Selected Area Electron Diffraction
SEM	Scanning Electron Microscopy
SPR	Surface Plasmon Resonance
